# Controlled Surface Modification of Polyamide 6.6 Fibres Using CaCl_2_/H_2_O/EtOH Solutions

**DOI:** 10.3390/polym10020207

**Published:** 2018-02-21

**Authors:** Barbara Rietzler, Thomas Bechtold, Tung Pham

**Affiliations:** Research Institute of Textile Chemistry and Textile Physics, Leopold-Franzens University Innsbruck, Höchsterstraße 73, 6850 Dornbirn, Austria; barbara.rietzler@uibk.ac.at (B.R.); thomas.bechtold@uibk.ac.at (T.B.)

**Keywords:** polyamide fibres, surface modification, calcium chloride, complexation, water sorption

## Abstract

Polyamide 6.6 is one of the most widely used polymers in the textile industry due to its durability; however, it has rather limited modification potential. In this work, the controlled surface modification of polyamide 6.6 fibres using the solvent system CaCl_2_/H_2_O/EtOH was studied. The effects of solvent composition (relative proportions of the three components) and treatment time on fibre properties were studied both in situ (with fibres in solvent) and ex situ (after the solvent was washed off). The fibres swell and/or dissolve in the solvent depending on its composition and the treatment time. We believe that the fibre–solvent interaction is through complex formation between the fibre carbonyl groups and the CaCl_2_. On washing, there is decomplexation and precipitation of the polymer. The treated fibres exhibit greater diameters and surface roughness, structural difference between an outer shell and an inner core is observable, and water retention is higher. The solvent system is more benign than current alternatives, and through suitable tailoring of the treatment conditions, e.g., composition and time, it may be used in the design of advanced materials for storage and release of active substances.

## 1. Introduction

Polyamide 6.6 (PA 6.6) is one of the most widely used polymers in the textile industry. Because of their outstanding mechanical properties, PA 6.6 fibres are used in apparel, in technical textiles, and as reinforcement in textile-based composites. PA 6.6 fibres show very smooth surfaces with low surface energies and are inert towards chemicals. It makes the fibres, in turn, less suitable for applications requiring high adhesion to other compounds or higher sorption capability. In such cases, surface treatment techniques are usually applied. Chemical and physical methods are commonly used to change surface properties such as wettability, biocompatibility, adhesive bonding, and dye sorption. Methods such as plasma treatment [1,2,3,4,5,6,7], chemical grafting [8,9,10], and enzymatic hydrolysis [11,12] are applied. For example, Li et al. [5] utilised atmospheric plasma to improve the hydrophilicity of PA 6.6 fabrics, depending on the discharge conditions. Other researchers [6] reported on better interfacial adhesion between the polyamide 6 (PA 6) matrix and polyurethane adhesive layer in polymer–carbon fibre composites by treating PA 6 with plasma. Zhao et al. [9] applied chemical grafting of 2-hydroxyethyl methacrylate onto PA 6.6 fabric, also to improve its hydrophilicity. A protease from the bacterium *Bacillus licheniformis* has been investigated as a biocatalyst for the modification of PA 6.6 fibres with respects to dyeability [11]. Another modification method is the formation of an acid–base complex between Lewis acids, such as GaCl_3_ and AlCl_3_, and the Lewis base sites, the carbonyl groups (C=O) in polyamides [13,14,15,16,17]. The use of CaCl_2_ with methanol as the mediator to form a complex with the PA 6.6 structure is also reported by other groups [18,19,20]. Li et al. used a mixture of CaCl_2_/EtOH to modify the surface properties of aromatic polyamide fibres (Kevlar), in order to improve the interfacial adhesion in fibre composites [21]. The integration of a Lewis acid into the polymer structure leads to modified crystallisation behaviours and therefore changes polymer properties [15,22,23].

The focus of this work was the modification of PA 6.6 fibres with special emphasis on the swelling of PA 6.6 fibres. This enables the modification of the physical surface properties such as roughness, sorption behaviour, and changes of the fibre diameter. The treatment was achieved by using solvents containing CaCl_2_/EtOH/H_2_O in different compositions. Compared to the already known solvents such as MeOH/CaCl_2_, phenols, and formic acid, these solutions are less toxic and less environmentally harmful. In situ and ex situ microscopic studies were performed in order to investigate the effect of solvent composition and the treatment time on the fibre modification. The modified PA 6.6 fibres were investigated using Fourier transform infrared spectroscopy (FTIR). The topography and line roughness of the formed surface layer were determined using confocal 3D laser-scanning microscopy. Furthermore, the liquid sorption capability of the modified fibres was characterised by water retention value (WRV) measurements.

## 2. Materials and Methods

### 2.1. Materials

Throughout this work, PA 6.6 filaments with circular cross-sections and an average diameter of 15.6 µm, kindly supplied from Schoeller GmbH & CoKG, Hard, Austria, were used as received. Calcium chloride dihydrate (analytical grade), ethanol (99.9% for analysis, Österreichische Agrar-Alkohol HandelsgesmbH., Spillern, Austria), and deionised water were used to prepare the treatment solvents.

### 2.2. Sample Preparation

#### 2.2.1. Mapping of Solvent Quality as a Function of Composition

Calcium chloride dihydrate was dissolved in absolute ethanol and deionised water. The exact composition was calculated from the weighted masses of all three components used for the preparation. Twenty-six different solvent compositions were tested (Table 1). The observations were performed on an object slide where the fibres were attached. A maximum of four fibres was attached to the surface of one object slide. The fibres were completely covered with an excess of solvent. To prevent evaporation, a coverslip was placed on top of it. The fibres were allowed to stay in the solvents for several hours at ambient temperature. During that time, the fibres were examined under an Olympus CX 41 optical light transmission microscope (Olympus Corporation, Tokyo, Japan) to observe whether the solvents dissolve, swell, or have no effect on the fibres. Based on the results of the observations, we mapped areas of “no effect” (area I), “dissolution” (area II), and “swelling” (area III) in a ternary phase diagram.

#### 2.2.2. In Situ Measurement

To investigate the effects of swelling and dissolution on the PA 6.6 fibres, one solvent representative of each of areas II and III was chosen. The swelling solvent (abbreviated SW) from area III was composed of 12.50 mol % CaCl_2_, 68.75 mol % H_2_O, and 18.75 mol % EtOH (Solvent 2, Table 1). The dissolving solvent (abbreviated DISS) from area II was composed of 12.50 mol % CaCl_2_, 25.00 mol % H_2_O, and 62.50 mol % EtOH (Solvent 17, Table 1). These solvents were the best swelling and dissolving solvents found in the previous experiment. All further experiments in this work were carried out with these two solvents. For the in situ measurement, one fibre was attached to an object slide and covered with an excess of solvent. Another shortened object slide was placed on top to prevent the evaporation of ethanol. The fibres were placed under an optical light transmission microscope of 10× magnification (Reichert Biovar Austria). Pictures were taken with a digital camera attached to the microscope. The diameter changes were documented in 5 min steps by recording images, and measurements were performed with image analysis using Adobe^®^ Photoshop^®^ CS 4 Extended. (Adobe Systems Incorporated, San Jose, CA, USA 2008). The results were the mean of five measurements for each solvent and the corresponding standard deviation.

#### 2.2.3. Measurement after Washing

After the fibres were soaked in SW and DISS for 5 to 60 min with 5 min steps, they were rinsed with deionized water to remove the solvent and examined under an Olympus CX 41 optical light transmission microscope (Olympus Corporation, Tokyo, Japan) at 40× magnification. The diameter measurements were performed with image analysis using the onboard software. The results are the mean of 20 measurements and the corresponding standard deviation.

#### 2.2.4. Fibre Bundle Treatments

A bundle of PA 6.6 fibres of 1 g was weighed and covered with 20 g of solvent for 3, 5, 7, and 10 min. The treatments were performed with the SW and DISS solvent. After the treatment, the fibre bundle was removed and immediately washed in excess deionized water. The complete removal of CaCl_2_ was ensured by titrimetric calcium analysis (data not given). The fibres were then allowed to dry.

### 2.3. Analytical Methods

#### 2.3.1. Fourier Transform Infrared Spectroscopy (FTIR)

The Fourier transform infrared spectra (FTIR) of the fibres were recorded using the attenuated total reflectance (ATR) unit equipped with a diamond crystal, using the FTIR spectrometer (Bruker Vector 22, Karlsruhe, Germany) with the spectral range 4000 to 500 cm^−1^, resolution 2 cm^−1^, and 128 scans.

#### 2.3.2. Water Retention Value Determination

The fibres were conditioned in a climate room with 20 ± 2 °C and 65% ± 4% RH for at least 24 h, after disentangling fibre bundles when necessary. They were then weighed (*m*_c_) and immersed in deionized water for 24 h. The soaking wet fibres were placed in filtration tubes (10 µm porosity) and centrifuged at 2500 G for 10 min in a laboratory centrifuge (Heraeus Multifuge, 1 L, Hanau, Germany). The fibres were reweighed immediately after centrifugation (*m*_w_) and the water retention value (WRV) was calculated as follows:

WRV = [(*m*_c_ − *m*_w_)/*m*_c_] × 100 (1)

#### 2.3.3. Confocal 3D Laser-Scanning Microscopy

The virgin PA 6.6 fibres and fibres washed after treatment with SW solvent for 10 min were examined with a confocal 3D laser-scanning microscope (Keyence VK-X150, Keyence, Japan). Their surface roughness was determined on images recorded at 100× magnification using the onboard software. The mean of five independent measurements is presented.

## 3. Results and Discussion

### 3.1. Swelling and Dissolution Behaviour of PA 6.6 Fibres in CaCl_2_/H_2_O/EtOH Solvents

For the qualitative observation of swelling and dissolution behaviour, 26 different compositions of CaCl_2_/EtOH/H_2_O were prepared and their effects on PA 6.6 fibres were investigated (Table 1).

**Figure 1:**
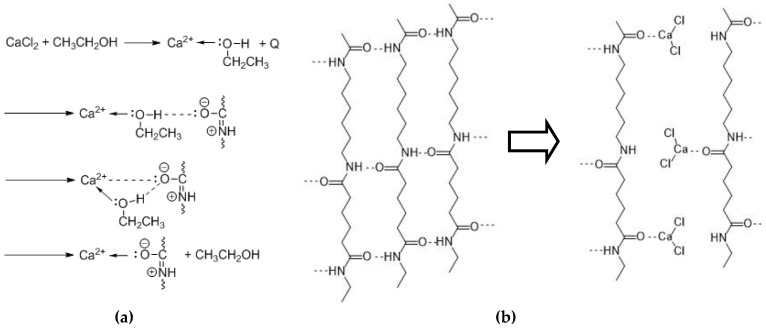
Proposed complexation mechanism of polyamide 6.6 (PA 6.6) in CaCl_2_/EtOH/H_2_O solvent: (**a**) Complex formation; (**b**) Disruption of hydrogen bonds in PA 6.6 in the presence of CaCl_2_ according to [24].

When PA 6.6 is added to the alcoholic solution, the carbonyl group approaches the calcium–alcohol complex. As the affinity of calcium is higher towards the oxygen of the carbonyl group than the hydroxyl, a transfer occurs. Thus, a complex between calcium and PA 6.6 forms and the alcohol becomes free again (Figure 1a). Due to the complex formation, hydrogen bonds are disrupted (Figure 1b) and dissolution of PA 6.6 takes place. The mechanism shown in Figure 1 has been proposed by Sun based on methanol/CaCl_2_ [24]. The concept of the disruption of the hydrogen bonds between the polyamide chains by Lewis acids has also been reported by other authors [13,14,15,18].

**Figure 2:**
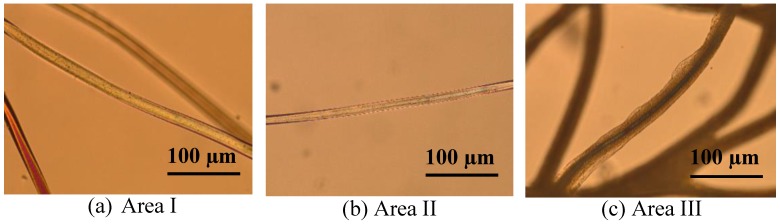
Pictures of the PA 6.6 fibres treated with solvents of the corresponding area: (**a**) No effect of the solvent on the fibres; (**b**) Dissolution of the fibre surface leading to fibre thinning (DISS); (**c**) Formation of a swollen shell (SW).

In this case, hydrolysis due to alkaline pH medium can be excluded because the formal pH measured in the concentrated solution was observed at 3.2, which is in agreement with the Lewis acid character of CaCl_2_. However, it must also be taken into consideration that the concept of pH is only valid in diluted aqueous solutions. In our case, we have concentrated ethanolic solutions, hence the pH value cannot be utilised to characterize the solution system. Essentially, the dissolution process in polymers is based on two different transport processes; diffusion and chain disentanglement [25]. The dissolution starts when the polymer comes into contact with a suitable solvent. Then the solvent starts to penetrate into the polymer and interactions between the polymer chains are broken; this is the diffusion process, which induces swelling. In the swollen polymer, the chains are still entangled, and the chain disentanglement proceeds at the interface between the swollen polymer and the solvent. Depending on the velocities of these two processes, either swelling or dissolution occurs. The photomicrographs in Figure 2 show the different behaviours: (a) no effect of the solvent on the fibres; (b) dissolution of the fibre surface leading to fibre thinning; and (c) formation of a swollen shell.

**Figure 3:**
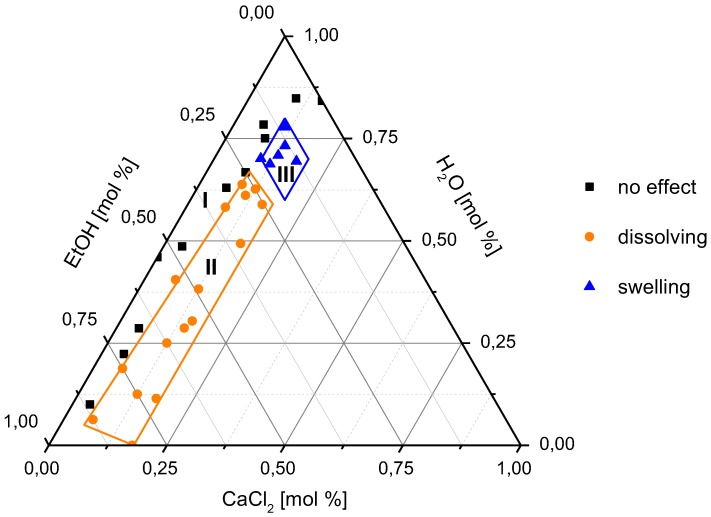
Ternary phase diagram of the CaCl_2_/H_2_O/EtOH system for PA 6.6 fibre treatment.

The observed results were plotted in a ternary phase diagram (Figure 3) with different symbols representing the behaviours of polyamide fibres in the different solvents.

It is reported in the literature that adding small amounts of non-solvent to a solvent changes the dissolving and swelling behaviour of a polymer [25]. In the case of the mixture of calcium chloride and ethanol, the addition of water caused an increase of the swelling rate of PA 6.6 fibres. It was found that not only the calcium chloride content plays an important role, but also does the amount of water, and therefore the ratio between water and ethanol has an impact. As a result, it was possible to separate the ternary phase diagram into three areas: I, II, and III; and they can be assigned to the different effects of the solvents (Figure 3, Table 2).

Solvents with CaCl_2_ content above 6 mol % and below 10 mol %, ethanol content lower than or equal to 25 mol %, and H_2_O/EtOH mole ratios above 2.5 did not dissolve the fibres. The same was observed for solvents with CaCl_2_ content below 6 mol %, ethanol content above 25 mol %, and H_2_O/EtOH mole ratio below 2.5. These values describe area I in Figure 3. Solvents with CaCl_2_ amount above 6 mol %, ethanol content above 25 mol %, and H_2_O/EtOH mole ratios below or equal to 2.5 dissolved the fibres. The solvents in area II caused a reduction of the fibre diameter and later complete dissolution. Solvents that caused swelling had CaCl_2_ amounts greater than 10 mol %, ethanol content below 25 mol %, and H_2_O/EtOH mole ratios greater than 2.5. In these swelling solvents, the outer diameter of the fibre increased. Under the microscope, a swollen outer shell and a core region were observed. The size of the shell region increased with time, while that of the core region decreased. Finally, the whole fibre was swollen and no core region was observed. This effect is referred to as area III.

In area II (dissolving), the diffusion velocity of the solvent into the polymer is the same or slower than the polymer chain disentanglement at the surface. In this case, no gel-like layer was observed because of the high velocity of the disentanglement.

In area III (swelling), the mechanism is different. The amounts of deionised water, the non-solvent in this case, were much higher. Water molecules are very small and mobile, thus diffuse easily into the polymer and cause swelling. However, the transport of polymer molecules from the surface into solution is slow. This is the reason that swelling was observed. Furthermore, the phenomena of swelling or dissolving depended on the water/ethanol mole ratio and the amount of CaCl_2_ in the solution. Although this effect had not been reported for PA 6.6 fibres, it has been observed for ramie fibres, cotton, kraft pulp, and rayon in *N*-methylmorpholine *N*-oxide containing various amounts of water [26,27].

### 3.2. Microscopic Investigation of Swelling and Dissolving of PA 6.6 Fibres

To quantify the previously described observations of swelling and dissolution, diameter measurements were conducted under the microscope. Two solutions were chosen to be investigated more thoroughly: one of the solutions with a H_2_O/EtOH mole ratio below or equal to 2.5 (dissolving solution 17 in Table 1); and the other with a H_2_O/EtOH mole ratio higher than 2.5 (swelling solution 2 in Table 1).

#### 3.2.1. In Situ Swelling and Dissolution Experiments

**Figure 4:**
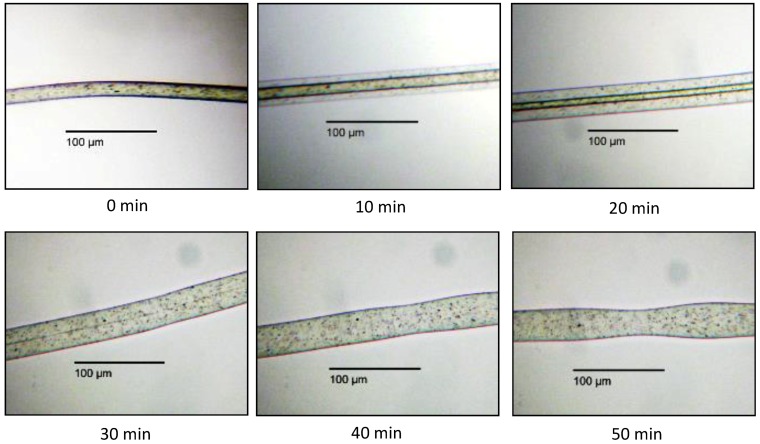
Swelling of PA 6.6 fibre in swelling solvent (SW).

At first, the fibre diameters were examined in situ according to the experimental procedure described above; therefore, no mechanical forces were affecting the fibres. In Figure 4, swelling of PA 6.6 fibres in SW is shown as a function of time.

The swelling started immediately after adding the solvent to the fibres. After 10 min, the outer diameter was already increased by 77.0%, and a further increase was observed until the core region disappeared. Note that the average diameter of the PA 6.6 fibres was initially around 15.6 µm. During the soaking in SW, the outer shell diameter increased while the inner core diameter decreased. After 20 min, the outer shell diameter had increased by 100.4%. Thereafter the swelling rate decreased. A table with the detailed values listed can be found in the Supplementary Information (Table S1). The picture after 50 min shows that the shape of the fibre was starting to become more irregular, which indicates that the fibre was losing its integrity with increased swelling. This can be explained by the decrease of interactions between the polymer chains, because the hydrogen bonds are severed as the complex between the carbonyl groups and the CaCl_2_ is formed (Figure 4). To obtain a reliable value of fibre diameter, it was measured once at the thicker site and at the thinner site and the mean value was calculated.

**Figure 5:**
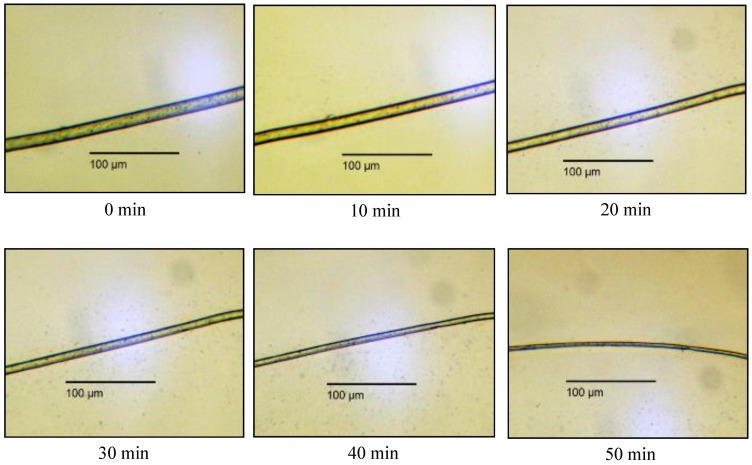
Dissolution of PA 6.6 fibre in dissolving solvent (DISS).

For comparison, pictures of fibres in DISS were also taken as a function of treatment time (Figure 5).

In this solvent, no gel-like layer was visible, but an attenuation was observed. After 20 min, the diameter was already decreased by 25.2% of the initial diameter. After 50 min, the diameter reached a width of around 4.3 µm. Afterwards, the fibre was not visible anymore and had dissolved completely. A table with the detailed values listed can be found in the Supplementary Information (Table S2). Thus, no swelling was occurring in this solvent, and instead the fibre was thinning until the whole fibre had dissolved.

**Figure 6:**
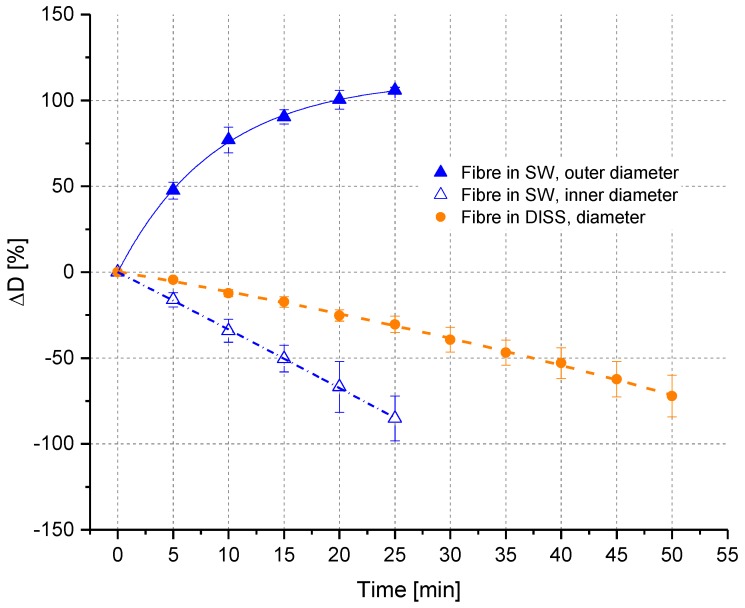
Fibre diameter changes (ΔD in %) depending on time in SW and DISS in in situ experiments.

**Figure 7:**
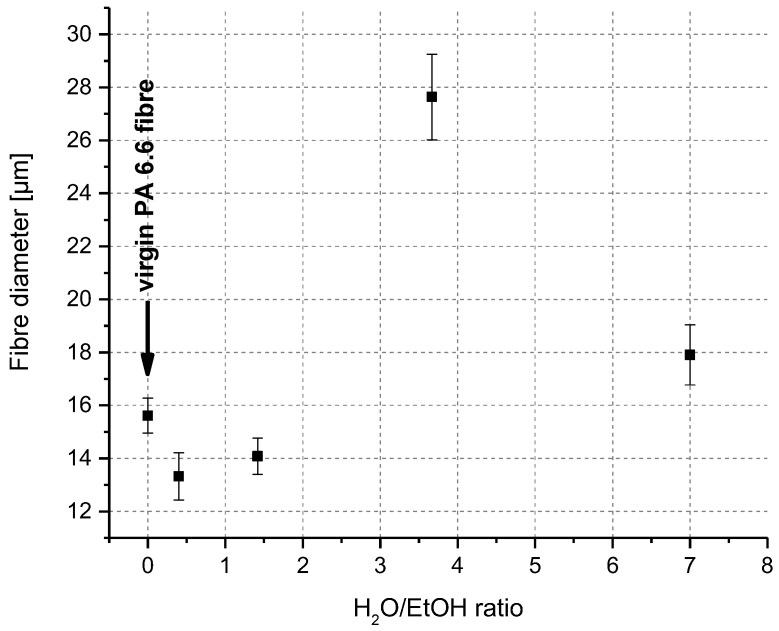
Fibre diameter as a function of H_2_O/EtOH ratios in CaCl_2_/H_2_O/EtOH mixtures after 10 min treatment.

In Figure 6, the diameter changes of fibres in SW and DISS are compared. In DISS, it was observed that the diameter was decreasing linearly over time. In the case of SW, a non-linear increase of the outer fibre diameter was noticed, accompanied by a decrease of the core diameter. The high swelling rate at the beginning was decreasing over time. When the fibre was completely swollen and no core was left, the swelling stopped. In Figure 7, the comparison of four solvents with different H_2_O/EtOH mole ratios for a treatment time of 10 min is shown.

An increased water amount and therefore higher H_2_O/EtOH mole ratios resulted in a higher swelling rate. The critical value of the H_2_O/EtOH mole ratio where swelling starts is 2.5. Below that value, no swelling was observed, but a higher dissolution rate was detected. These observations support the dissolution mechanism of polymers as described above. As seen in Figure 7, the swelling rate did not increase continuously with an increasing H_2_O/EtOH ratio, and it decreased again at high water amounts. The explanation for this phenomenon could be visualised using the ternary phase diagram (Figure 3). With increasing H_2_O/EtOH ratios, the solvent composition was shifted along the ethanol axes, from area II (dissolving leading to smaller fibre diameter) to area III (swelling leading to bigger fibre diameter). However, with further increase of the water content, the solvent composition was further shifted close to area I (no effect). In this case, water started to act as a coagulating agent and thus prevented further diffusion of PA 6.6 chains into the solvent medium. Therefore, a smaller increase in fibre diameter was observed (Figure 7).

#### 3.2.2. Ex Situ Swelling and Dissolution Experiments with a Washing Step

In further experiments, the fibres were treated for definite time periods and then washed with deionised water to remove the solvent. In order to identify possible chemical changes, the fibres were characterised by infrared spectroscopy.

**Figure 8:**
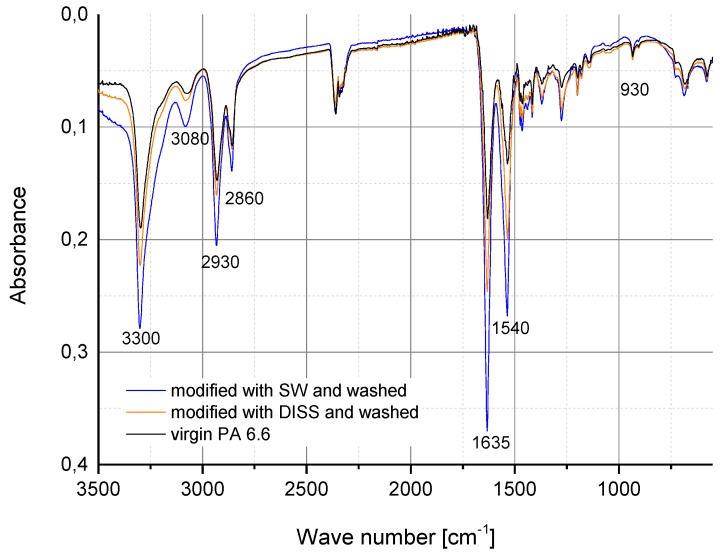
FTIR spectra of virgin PA 6.6 fibre and modified fibres using SW and DISS respectively after washing.

In Figure 8, FTIR spectra of virgin fibres and modified fibres using SW and DISS after the washing step with deionised water are shown. It can be observed that all spectra are very similar, showing characteristic peaks of PA 6.6. When CaCl_2_ was removed by washing, decomplexation of the PA 6.6–CaCl_2_ complex took place and the dissolved polymer was precipitated [23]. FTIR spectra in Figure 8 indicated that there were no changes in the chemical structure of PA 6.6. fibres. In other words, the decomplexation led to an intact PA 6.6 structure, comparable to the virgin one before the treatment. Sun reported a shift of the amide I band to lower frequencies in the FTIR spectra after forming a complex between polyamide and the CaCl_2_ [24]. They evaporated the solvent after treatment and thus CaCl_2_ residues were expected to remain on the substrate. In our study, the fibres were extensively washed with deionised water to remove solvent residues. Representative specimens analysed for calcium with titrimetric determination showed no detectable amounts (data not given). Hence, the FTIR spectra in this study indicate the complete decomplexation of the polyamide after precipitation and washing with deionised water.

In the case of the fibre in SW, a swollen shell was observed under the light microscope, which appeared to be in an equilibrium state of neither dissolved nor undissolved PA 6.6 adhering to the surface of the bulk PA 6.6 of the core region. This shell was returning to an undissolved state after removing the solvent by washing with deionised water. Observations under the microscope after washing still showed the swollen shell and the core region. The completely dissolved proportion of PA 6.6 was precipitating in the form of small particles, which caused turbidity of the solution in the washing step. Furthermore, the treated and washed fibres exhibited a rougher surface all over the fibre compared to the untreated fibres. In the case of the fibres in DISS, a swollen shell was not observable under the light microscope and thus it appears that dissolved PA 6.6 was dispersed in the solvent. With the addition of water, the dissolved PA 6.6 also precipitated in the form of small particles. The turbidity of the solution was much higher because of the precipitation. The fibres still had a smooth surface but some of the precipitated particles were retained at the surface of the treated fibres.

**Figure 9:**
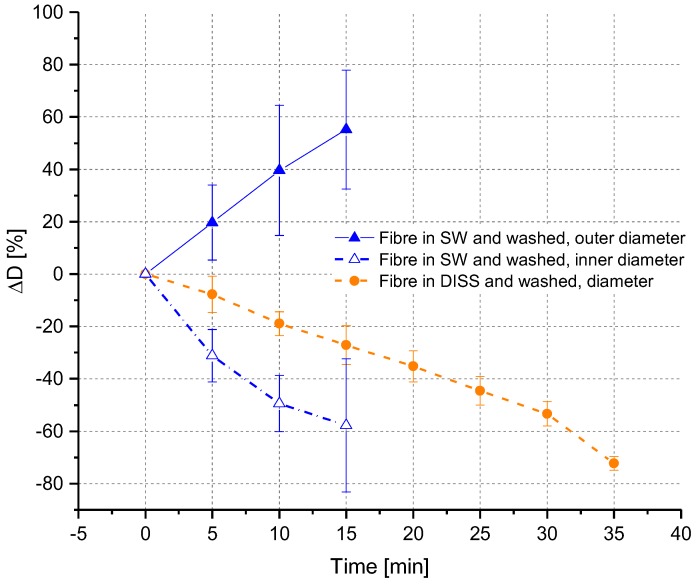
Changes in fibre diameter over time after washing.

In Figure 9, the diameter changes vs time are illustrated for the fibres in DISS and in SW after the washing step. For the fibres in SW, measurements were carried out until a treatment time of 15 min. After longer treatment times, the fibre strength was too low to withstand the washing procedure and the fibres broke or deformed, and therefore no diameter measurements were possible. In DISS, measurements were only possible until a treatment time of 35 min. Tables with the detailed values listed can be found in the Supplementary Information (Tables S3 and S4).

### 3.3. Liquid Storage Capability

With the determination of the water retention value, the ability of fibres to absorb and retain water is examined. To study the effect of the modification on the water retention value, a PA 6.6 fibre bundle was modified with SW and DISS as described in Section 2.2.4.

**Figure 10:**
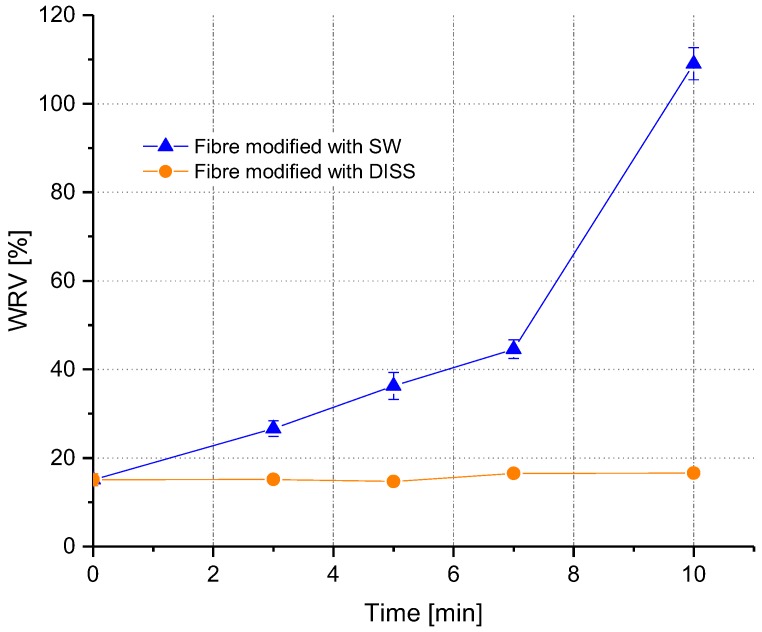
Water retention value vs time of treatment using DISS and SW.

Figure 10 shows the water retention values (WRV) of the modified fibres after different treatment times.

**Figure 11:**
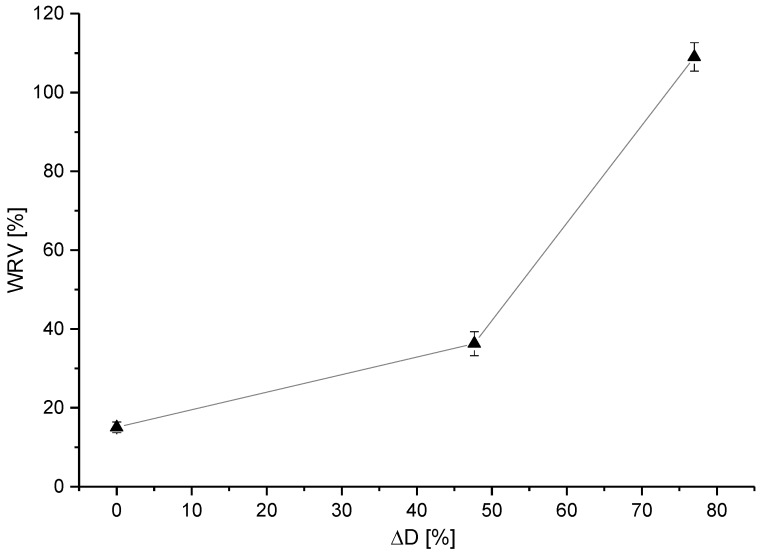
Water retention value vs diameter change in percentage after 10 min treatment with SW.

The water retention value for the fibres treated with SW increased considerably with longer treatment times. In contrast to this, the water retention value did not change significantly for the fibres treated with DISS. The fibres treated with DISS showed WRV values between 14% and 16%, whereas the virgin fibres exhibited a WRV of around 15%. After 3 min of treatment with SW, the WRV rose to ca. 26%, much higher than the WRV of the virgin fibres (Figure 10). After 10 min, the WRV reached a value of ca. 109%. This indicates that the water retention value is well correlated to the size of the swollen shell; as with longer treatment times, the shell size was also increasing (Figure 11).

### 3.4. Surface Analysis by 3D Confocal Laser-Scanning Microscopy

**Figure 12:**
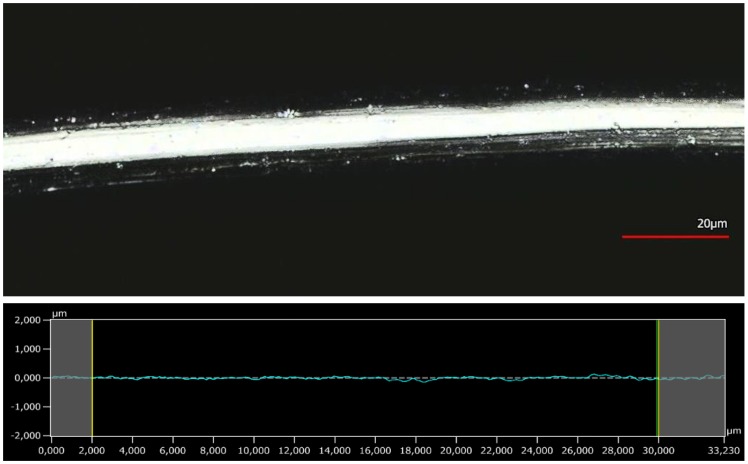
3D confocal laser-scanning microscope picture of untreated PA 6.6 fibre and roughness profile.

**Figure 13:**
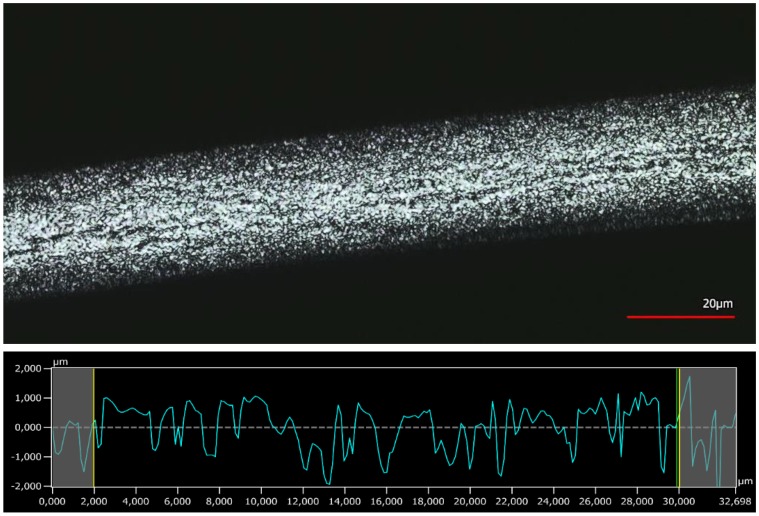
3D confocal laser-scanning microscope picture of PA 6.6 fibre modified using SW and roughness profile.

The surface profiles of a virgin and modified fibre are shown in Figure 12 and Figure 13, respectively. The top half in each figure shows the surface, and the bottom half the roughness profile. The roughness profile is used in determinations of the Ra (roughness parameter), which is the arithmetic average of the vertical deviations along the surface profile.

As seen in Figure 12, the surface of the untreated fibre is very smooth; as seen in Figure 13, the modified fibre surface is very rough. The corresponding Ra values were 0.083 ± 0.037 µm and 0.465 ± 0.059 µm, respectively. This finding supports the proposed mechanism of dissolution and swelling. PA 6.6 in the equilibrium state is reprecipitating unevenly and therefore introduces a high level of roughness onto the fibre surface. In addition to the swollen shell structure, the higher roughness of the modified fibre may also contribute to the increased water retention value. However, it is difficult to assess the relative influence of the two factors.

## 4. Conclusions

In this study, we investigated the effects of CaCl_2_/EtOH/H_2_O on PA 6.6 fibres. Through complexation, the PA 6.6 can be dissolved. Depending on the relative amounts of these three components, the fibre–solvent interaction is different. It was found that the molar ratio between water and ethanol and the amount of calcium chloride in the mixtures are key parameters. In solvents with higher water amounts relative to ethanol, the fibres swell considerably [28]. On the contrary, in solvents with higher ethanol amount and less water, the fibres are dissolved without the formation of a visible gel-like layer. The treatment increased fibre swelling and surface roughness, and the modified fibres showed a higher water retention. Further experiments on the sorption behaviour will be carried out to study the modification process in more detail. This solvent system, CaCl_2_/EtOH/H_2_O, is more benign than other known solvents for PA 6.6. It can be applied for defined PA 6.6 fibre surface modification with different degrees of modification, but also for selective PA 6.6 fibre dissolution and coagulation, e.g., in PA 6.6 fibre recycling. Through the introduction of a higher surface roughness by controlled surface modification and the swollen shell structure, the modified fibres can be utilised for storage and release of active substances.

## Figures and Tables

**Table 1:** Compositions of different solvents for the dissolving and swelling observation.

Sample Number	CaCl_2_ (mol %)	H_2_O (mol %)	EtOH (mol %)	H_2_O/EtOH Mole Ratio	Observation
1	10.00	70.00	20.00	3.50	swelling
2	12.50	68.75	18.75	3.67	swelling
3	13.33	73.33	13.33	5.50	swelling
4	17.16	62.13	20.71	3.00	swelling
5	18.00	69.50	12.50	5.56	swelling
6	11.11	77.78	11.11	7.00	swelling
7	8.33	58.33	33.33	1.75	dissolving
8	12.50	56.25	31.25	1.80	dissolving
9	12.50	62.50	25.00	2.50	dissolving
10	15.20	30.40	54.40	0.56	dissolving
11	15.97	49.28	34.75	1.42	dissolving
12	6.64	40.37	52.98	0.76	dissolving
13	16.50	27.00	56.50	0.48	dissolving
14	12.50	12.50	75.00	0.17	dissolving
15	19.00	12.50	68.50	0.18	dissolving
16	6.25	18.75	75.00	0.25	dissolving
17	12.50	25.00	62.50	0.40	dissolving
18	8.33	75.00	16.67	4.50	no effect
19	8.33	66.67	25.00	2.67	no effect
20	10.00	85.00	5.00	17.00	no effect
21	6.36	78.32	15.32	5.11	no effect
22	3.94	48.57	47.49	1.02	no effect
23	0.00	46.01	53.99	0.85	no effect
24	4.63	28.50	66.88	0.43	no effect
25	4.80	22.30	72.90	0.31	no effect
26	3.70	10.00	86.30	0.12	no effect

**Table 2:** Description of the areas assigned to the three different effects.

Effect of Solvent	CaCl_2_ (mol %)	EtOH (mol %)	H_2_O/EtOH Mole Ratio
No effect (area I)	6 < x < 10	x ≤ 25	x > 2.5
No effect (area I)	x < 6	x > 25	x < 2.5
Dissolving (area II)	x > 6	x > 25	x ≤ 2.5
Swelling (area III)	x > 10	x < 25	x > 2.5

## Supplementary Materials

The following are available online at http://www.mdpi.com/2073-4360/10/2/207/s1, Table S1: Results of diameter measurements and the changes of the diameter in % for PA 6.6 fibres in SW; Table S2: Results of diameter measurements and the changes of the diameter in % for PA 6.6 fibres in DISS; Table S3: Results of diameter measurements and the changes of the diameter in %.for PA 6.6 fibres in SW after washing; Table S4: Results of diameter measurements and the changes of the diameter in %.for PA 6.6 fibres in DISS after washing.
