# Supplementary material for: Controlled Surface Modification of Polyamide 6.6 Fibres Using CaCl2/H2O/EtOH Solutions
Source: Polymers (Basel). 2018 Feb 21;10(2):207. doi: 10.3390/polym10020207 (PMC6415128; doi:10.3390/polym10020207)
Supplement: Supplementary file 1 [file polymers-10-00207-s001.pdf]

Controlled surface modification of polyamide 6.6 fibres using $CaCl_2/H_2O/EtOH$ solutions

Barbara Rietzler, Thomas Bechtold, Tung Pham*

Research Institute of Textile Chemistry and Textile Physics, Leopold-Franzens University Innsbruck, Höchsterstraße 73, 6850 Dornbirn, Austria; textilchemie@uibk.ac.at

* Correspondence: tung.pham@uibk.ac.at; Tel.: +43 5572 28533 0

Supplementary Materials:

Table S1: Results of diameter measurements and the changes of the diameter in % for PA 6.6 fibres in SW; D_o – outer diameter, D_c – diameter of core, σ – standard deviation, Δ – difference of diameter at the beginning and after the treatment (in-situ experiments)

Time [min]	D_o [µm]	σD_o [µm]	D_c [µm]	σD_c [µm]	ΔD_o [%]	$\sigma \Delta D_o$ [%]	ΔD_c [%]	$\sigma \Delta D_c$ [%]
0	15.6	0.8						
5	23.1	1.7	13.1	0.7	47.5	4.9	-16.1	4.2
10	27.6	1.6	10.3	1.2	77.0	7.5	-34.2	6.7
15	29.7	1.6	7.7	1.1	90.4	4.2	-50.4	7.7
20	31.3	1.6	5.2	2.3	100.4	5.4	-66.8	14.8
25	32.1	1.4	3.9	1.1	105.9	1.7	-85.2	13.0

Table S2: Results of diameter measurements and the changes of the diameter in % for PA 6.6 fibres in DISS; D_o – outer diameter, D_c – diameter of core, σ – standard deviation, Δ – difference of diameter at the beginning and after the treatment (in-situ experiments)

Time [min]	D [µm]	σ D [µm]	ΔD [%]	σ ΔD [%]
0	15.6	0.8	0.0	0.0
5	14.5	0.8	-4.3	1.2
10	13.3	0.9	-12.1	1.7
15	12.6	1.0	-17.2	2.8
20	11.3	0.9	-25.2	2.9
25	10.6	1.1	-30.2	4.2
30	9.2	1.5	-39.0	6.5
35	8.1	1.4	-46.6	6.6
40	7.2	1.6	-52.7	8.1
45	5.8	1.7	-62.0	9.3
50	4. 3	2.0	-71.7	11.1

Table S3: Results of diameter measurements and the changes of the diameter in %.for PA 6.6 fibres in SW after washing; D_o – outer diameter, D_c – diameter of core, σ – standard deviation, Δ – difference of diameter at the beginning and after the treatment (ex-situ experiments)

Time [min]	Do [µm]	σD_o [µm]	D_c [µm]	σD_c [µm]	ΔD_o [%]	$\sigma \Delta D_o$ [%]	ΔD_c [%]	$\sigma \Delta D_c$ [%]
0	15.7	0.4	15.7	0.4	0.0	0.0	0.0	0.0
5	18.7	2.3	10.8	1.6	19.7	14.3	-31.2	-10.0
10	21.9	3.9	7.9	1.7	39.6	24.8	-49.4	-10.8
15	24.3	3.6	6.6	4.0	55.2	22.7	-57.8	-25.4

Table S4: Results of diameter measurements and the changes of the diameter in %.for PA 6.6 fibres in DISS after washing; D – diameter, σ – standard deviation, Δ – difference of diameter at the beginning and after the treatment (ex-situ experiments)

Time [min]	D [µm]	σ D [µm]	ΔD [%]	σΔD [%]
0	15.7	0.4	0.0	0.0
5	14.5	1.1	-7.7	6.9
10	12.7	0.7	-18.9	4.6
15	11.4	1.2	-27.1	7.4
20	10.2	1,0	-35.2	5.9
25	8.7	0.9	-44.6	5.5
30	7.3	0.7	-53.3	4.6
35	4.3	0.4	-72.3	2.6
